# Risk stratification using Artificial Intelligence: Could it be useful to reduce the burden of chronic kidney disease in low- and middle-income Countries?

**DOI:** 10.3389/fpubh.2022.999512

**Published:** 2022-09-29

**Authors:** Angela J. Pereira-Morales, Luis H. Rojas

**Affiliations:** ^1^PhD Program in Public Health, School of Medicine, Universidad Nacional de Colombia, Bogotá, Colombia; ^2^Science for Life (S4L), 10x Research Group, Bogotá, Colombia

**Keywords:** Risk stratification - ARVC/D, CKD - Chronic kidney disease, Artificial intelligence (AI), machine learning (ML), Prevention

## Introduction

Chronic kidney disease (CKD) is an important public health burden, with a marked increase in morbidity and mortality as well as a huge economic burden worldwide (1). Currently, the economic burden of CKD in low- and middle-income countries (LMICs) is high and is significant mainly for stages 4 to 5 of the disease (2), with more than 60% of the global burden of CKD occurring in LMICs (1).

In recent decades the prevalence of diabetes and hypertension has increased worldwide, but at a faster rate in LMICs (3). This is relevant considering the role of diabetes and hypertension as the main precursors of CKD, and the fact that the onset and progression of CKD, and cardiovascular consequences, might be reduced through prevention, early detection, and intensive control of glycemia and hypertension.

For clinicians and health care professionals it is well known that CKD patients have a high heterogeneity in disease manifestation, progression, and response to treatment. Furthermore, CKD is known to be complex and highly multifactorial, often with late diagnosis and chronic progression. Artificial intelligence (AI) can make significant contributions to the understanding of the course of CKD for more precise phenotype and outcome prediction (4).

## AI and health care

The most suitable definition for AI could be the engineering of creating intelligent machines with the capability to resolve many tasks by taking advantage of the huge available amounts of data (5). An enormous amount of data from healthcare systems in LMICs is now available and usable. With the continued expansion of digital data in all aspects of healthcare AI can be used for disease diagnosis, prognosis, and other risk prediction (6).

One of the main branches of AI is machine learning (ML), a discipline dedicated to the study of algorithms and the application of statistical models to learn from the past, aiming to identify patterns and continually increasing experience to understand the complexity of the problem and making predictions based on the story told by the data (7).

It has been found that in many countries, including LMICs, fewer than 15% of patients with CKD are aware that they have this condition; moreover, treatment gaps range from 68.7 to 97.8% (8). These findings may suggest that systematic screening and subsequent risk stratification for CKD could be associated with both clinical and public health benefits.

Evidence on the cost of implementing machine learning-based algorithms for CKD has reported that the savings for healthcare systems could be significant. For example, a study estimated the budgetary impact in the United States of implementing an AI-based risk stratification system for patients with type 2 diabetic kidney disease (stages 1–3b). The overall result is that the undiscounted savings in the 5-year base case for 100,000 patients tested with the system were $1.052 billion, mainly attributed to slowing disease progression (9).

## Risk stratification using AI for CKD management

Machine learning-based predictive models have demonstrated their ability to outperform risk calculators developed using conventional statistical methods for cardiovascular disease events and comorbidities such as diabetes and hypertension (10), demonstrating their potential to improve risk prediction and aid medical decision-making.

If early detection of patients with a higher risk of CKD is desired, previous evidence on the implementation of machine learning algorithms to stratify risk of CKD suggests that this could be more efficient and cost-effective than traditional population-based screening methods (9, 11, 12). Furthermore, risk stratification could be associated with a significant decrease in the number of individuals who required closer monitoring of the glomerular filtration rate (eGFR) and an increase in the proportion of patients for whom a treatment change is indicated. Therefore, a positive impact on the incidence and prevalence of CKD in patients with diabetes and hypertension can be expected together with a favorable cost-utility. Table 1 shows the rationale of risk stratification based on ML algorithms for early identification of at-risk patients and the existing interventions from primary to tertiary prevention.

**Table 1:** Rationale of the machine learning-based risk stratification system and target population.

	**General population**	**Newly diagnosed patients**	**Intermediate stage patients**	**Patient at high risk of needing dialysis in the next year**
***Population group***	Undiagnosed with risk factors	Initial stages (1–2)	Stages 1 to 3a	Stages 3b−4
***Prediction target***	Screening to identify individuals at risk of CKD diagnosis in the next year	Individual risk stratification for CKD accelerated progression at 6 and 12 months.	Individual risk stratification for accelerated CKD progression at 6 and 12 months.	Individual risk stratification for need for dialysis initiation in the next year
***Intervention***	Primary prevention (lifestyle modifications; dietary counseling; preventing and controlling obesity; improving blood glucose and blood pressure control)	Secondary prevention (control proteinuria; identify and provide effective pharmacotherapy; individualize therapy; identify and manage additional risk factors)	Secondary and tertiary prevention	Tertiary prevention (control uremic symptoms and comorbidities; control fluid and sodium retention; control cardiovascular risk factors; explore other supportive therapies and kidney preservation)
***Prediction based on ML***	High probability of developing: Diabetes Hypertension CKD	Low, medium, and high risk of CKD accelerated progression at 6 months and 1 year.	Low, medium, and high risk of accelerated CKD progression at 6 months and 1 year.	High probability of needing dialysis within 1 year

From a preventive point of view, identifying the subset of healthy patients, but with risk factors for the development of the main precursor diseases of CKD, and the subset of patients with mild to moderate CKD who subsequently progress rapidly in the short term, could have important clinical and economic implications, since these patients could benefit from a differential follow-up and intervention given modifiable risk factors, even from primary health care.

Currently, although risk management for CKD in LMICs is based on the detection of diseases that are possible causes of CKD (such as diabetes and hypertension) and the identification of CKD in early stages (13), interventions continue to arrive late because there are no systems in place that allow early identification of patients at increased risk or accurate prediction of relevant outcomes.

## Discussion

In the last 10 years, hypertension and diabetes, which are important comorbidities for CKD, are showing accelerated incidence and prevalence in developing countries (14).

Although there is existing evidence about the precision achieved using ML in the identification of at-risk populations for CKD, there is much more to be done to tap the potential of AI and observe real impacts on public health. Likewise, with the advent of real-world evidence and data collected from patients daily, it could further advance the development of more accurate and transparent risk stratification algorithms for CKD.

It is known that current stratification tools fail to characterize the risk of CKD rapid progression, mainly because they do not use longitudinal data of predictors usually measured in health care models, which leads, on the one hand, to the inability to capture the variability of the data and, on the other hand, to the fact that these tools have poor clinical usability (15, 16). Likewise, translating adequate risk stratification into clinical practice requires assessing the rate of decline in GFR of patients based on their own measurement history, and those who progress rapidly should receive prompt and aggressive treatment of modifiable risk factors and closer follow-up to mitigate future harm.

There is no debate about that mainly in LMICs the unresolved structural problems in health systems, the lack of evidence-based technology developments and idiosyncrasies made it difficult to implement chronic disease preventive models based on AI. Moreover, insufficient research about the need and impact of such adoptions can be considered a fundamental part of the problem.

The growing public health burden of CKD as well as the increase of its major precursor diseases, make it necessary to overcome those barriers and move forward in conducting evaluations to assess the potential impact of implementing AI-based technologies for risk stratification.

If the reported evidence is correct, such implementations could also improve the efficiency of health systems and reduce health inequities.

## Author contributions

AP-M designed and wrote the document. LR co-designed the document and revised the final manuscript. All authors contributed to the article and approved the submitted version.

## Conflict of interest

Authors AP-M and LR were employed by S4L.

## Publisher's note

All claims expressed in this article are solely those of the authors and do not necessarily represent those of their affiliated organizations, or those of the publisher, the editors and the reviewers. Any product that may be evaluated in this article, or claim that may be made by its manufacturer, is not guaranteed or endorsed by the publisher.
